# Intestinal Toxemia Botulism in 3 Adults, Ontario, Canada, 2006–2008

**DOI:** 10.3201/eid1801.110533

**Published:** 2012-01

**Authors:** Yolanda D. Sheppard, Dean Middleton, Yvonne Whitfield, Felix Tyndel, Shariq Haider, Jamie Spiegelman, Richard H. Swartz, Mark P. Nelder, Stacey L. Baker, Lisa Landry, Ross MacEachern, Sherri Deamond, Lorrie Ross, Garth Peters, Michelle Baird, David Rose, Greg Sanders, John W. Austin

**Affiliations:** Ministry of Health and Long-Term Care, Toronto, Ontario, Canada (Y.D. Sheppard, Y. Whitfield, M.P. Nelder, S.L. Baker);; Ontario Agency for Health Protection and Promotion, Toronto (D. Middleton);; Scarborough Hospital–Birchmount, Toronto (F. Tyndel);; McMaster University Medical Center, Hamilton, Ontario, Canada (S. Haider);; Humber River Regional Hospital, Toronto (J. Spiegelman);; Sunnybrook Health Sciences Centre, Toronto (R.H. Swartz);; Public Health Agency of Canada, Guelph, Ontario, Canada (L. Landry);; Durham Region Health Department, Whitby, Ontario, Canada (R. MacEachern, S. Deamond);; Niagara Region Public Health, Thorold, Ontario, Canada (L. Ross);; Toronto Public Health, Toronto (G. Peters);; Hamilton Public Health Services, Hamilton (M. Baird);; The Scarborough Hospital, Toronto (D. Rose);; Health Canada, Ottawa, Ontario, Canada (G. Sanders, J.W. Austin)

**Keywords:** intestinal botulism, Crohn disease, Clostridium botulinum, bacteria, adult intestinal toxemia botulism, Canada

## MEDSCAPE CME

Medscape, LLC is pleased to provide online continuing medical education (CME) for this journal article, allowing clinicians the opportunity to earn CME credit.

This activity has been planned and implemented in accordance with the Essential Areas and policies of the Accreditation Council for Continuing Medical Education through the joint sponsorship of Medscape, LLC and Emerging Infectious Diseases. Medscape, LLC is accredited by the ACCME to provide continuing medical education for physicians.

Medscape, LLC designates this Journal-based CME activity for a maximum of 1 *AMA PRA Category 1 Credit(s)*^TM^. Physicians should claim only the credit commensurate with the extent of their participation in the activity.

All other clinicians completing this activity will be issued a certificate of participation. To participate in this journal CME activity: (1) review the learning objectives and author disclosures; (2) study the education content; (3) take the post-test with a 70% minimum passing score and complete the evaluation at www.medscape.org/journal/eid; (4) view/print certificate.

**Release date: December 22, 2011; Expiration date: December 22, 2012**

## Learning Objectives

Upon completion of this activity, participants will be able to:

- Evaluate the epidemiology and microbiology of adult intestinal toxemia botulism.
- Analyze risk factors for adult intestinal toxemia botulism.
- Assess the clinical presentation of adult intestinal toxemia botulism.
- Evaluate the prognosis of adult intestinal toxemia botulism.

## CME Editor

**Carol E. Snarey, MA,** Technical Writer/Editor, *Emerging Infectious Diseases. Disclosure: Carol E. Snarey, MA, has disclosed no relevant financial relationships.*

## CME Author

**Charles P. Vega, MD**, Health Sciences Clinical Professor; Residency Director, Department of Family Medicine, University of California, Irvine. *Disclosure: Charles P. Vega, MD, has disclosed no relevant financial relationships.*

## Authors

*Disclosures:* ***Yolanda D. Sheppard, MPH; Dean Middleton, BSc, DVM, MSc; Yvonne Whitfield; Felix Tyndel, MD; Shariq Haider, MD, FRCPC; Jamie Spiegelman, MD, FRCP; Mark P. Nelder, PhD; Stacey L. Baker; Lisa Landry, BSc; Ross MacEachern; Sherri Deamond, MHSc; Lorrie Ross, PhD; Garth Peters; Michelle Baird, BSc, CPHI(C), CIC; David Rose, MD; Greg Sanders, BSc;*** *and* ***John W. Austin, PhD,*** *have disclosed no relevant financial relationships.* ***Richard H. Swartz, MD, PhD,*** *has disclosed the following relevant financial relationships: received grants for clinical research from Roche; served as an advisor or consultant for Bristol-Myers Squibb Company; served as a speaker or a member of a speakers bureau for Bristol-Myers Squibb Company.*
