# Supplementary material for: Intestinal Toxemia Botulism in 3 Adults, Ontario, Canada, 2006–2008
Source: Emerg Infect Dis. 2012 Jan;18(1):1–6. doi: 10.3201/eid1801.110533 (PMC3310098; doi:10.3201/eid1801.110533)
Supplement: Related texts

# Review article: Intestinal Toxemia Botulism in 3 Adults, Ontario, Canada, 2006–2008

**DOI:** 10.3201/eid1801.110533

**Authors:** Yolanda D. Sheppard, Dean Middleton, Yvonne Whitfield, Felix Tyndel, Shariq Haider, Jamie Spiegelman, Richard H. Swartz, Mark P. Nelder, Stacey L. Baker, Lisa Landry, Ross MacEachern, Sherri Deamond, Lorrie Ross, Garth Peters, Michelle Baird, David Rose, Greg Sanders, John W. Austin

**Affiliations:** Ministry of Health and Long-Term Care, Toronto, Ontario, Canada (Y.D. Sheppard, Y. Whitfield, M.P. Nelder, S.L. Baker);; Ontario Agency for Health Protection and Promotion, Toronto (D. Middleton);; Scarborough Hospital–Birchmount, Toronto (F. Tyndel);; McMaster University Medical Center, Hamilton, Ontario, Canada (S. Haider);; Humber River Regional Hospital, Toronto (J. Spiegelman);; Sunnybrook Health Sciences Centre, Toronto (R.H. Swartz);; Public Health Agency of Canada, Guelph, Ontario, Canada (L. Landry);; Durham Region Health Department, Whitby, Ontario, Canada (R. MacEachern, S. Deamond);; Niagara Region Public Health, Thorold, Ontario, Canada (L. Ross);; Toronto Public Health, Toronto (G. Peters);; Hamilton Public Health Services, Hamilton (M. Baird);; The Scarborough Hospital, Toronto (D. Rose);; Health Canada, Ottawa, Ontario, Canada (G. Sanders, J.W. Austin)

## Abstract

Five cases of intestinal toxemia botulism in adults were identified within an 18-month period in or near Toronto, Ontario, Canada. We describe findings for 3 of the 5 case-patients. Clinical samples contained *Clostridium* *botulinum* spores and botulinum neurotoxins (types A and B) for extended periods (range 41–61 days), indicative of intestinal toxemia botulism. Patients’ clinical signs improved with supportive care and administration of botulinum antitoxin. Peanut butter from the residence of 1 case-patient yielded *C*. *botulinum* type A, which corresponded with type A spores found in the patient’s feces. The food and clinical isolates from this case-patient could not be distinguished by pulsed-field gel electrophoresis. Two of the case-patients had Crohn disease and had undergone previous bowel surgery, which may have contributed to infection with *C. botulinum*. These cases reinforce the view that an underlying gastrointestinal condition is a risk factor for adult intestinal toxemia botulism.

Botulism is a neuroparalytic condition, typified by cranial nerve palsies that may be followed by descending symmetrical flaccid paralysis, which can lead to respiratory arrest and, in some instances, death (*1*). Intestinal toxemia botulism is an infectious form of botulism in which illness results from ingesting spores, which is followed by spore germination and intraluminal production of botulinum neurotoxins over an extended period (*2*). Intestinal toxemia botulism is rarely reported in adults; <20 cases have been described in the literature since the first reports in 1980 and 1981 (*3**,**4*). The rarity of this condition contributes to its obscurity, an obscurity that may negatively affect those infected through a lack of rapid diagnosis and treatment. Countries with published case reports of intestinal toxemia botulism in adults are the United States (*3**–**10*), Italy (*11**,**12*), Iceland (*9*), and Japan (*13*). The etiologic agents involved in intestinal toxemia botulism in adults are *Clostridium botulinum* types A, B, and F (*6**–**9**,**13**,**14*) and, more rarely, *C. baratii* type F (*10*), and *C. butryicum* type E (*11**,**12*).

Although considered a rare condition, intestinal toxemia botulism was reported in 5 adults in or near Toronto, Ontario, Canada, during November 2006–May 2008. We report the clinical, laboratory, and epidemiologic findings for 3 of the 5 case-patients. Details pertaining to the clinical course of illness of 1 of the patients were previously published in a case report (*15*). In this case series, we document 3 cases of adult intestinal toxemia botulism, which occurred in a small geographic area in a short period, and provide further evidence for the potential role of host factors, such as Crohn disease, previous bowel surgery, and short bowel syndrome, which may predispose a person to intestinal toxemia botulism.

We reviewed case investigation files from the Ontario Ministry of Health and Long-Term Care and the local public health units in or near Toronto. Physicians involved in the cases provided clinical summaries. Consent to publish information pertaining to the diagnosis of intestinal toxemia botulism was obtained from the 3 case-patients. Two additional cases were reported in November 2006 and May 2008; however, consent was not obtained from those patients.

Serum, stool, gastric aspirate, and enema samples were assayed for botulinum toxin by using the mouse bioassay (*16*). Stool and enema specimens were cultured for *C. botulinum* as described (*16*). Botulinum neurotoxin was identified by neutralization with specific antitoxin types A, B, E, and F obtained from the Centers for Disease Control and Prevention (Atlanta, GA, USA). *C. botulinum* was identified and differentiated from other botulinum toxin–producing clostridia on the basis of Gram stain reaction, morphologic features, motility, lipase and lecithinase reactions on egg yolk agar, sugar fermentations, esculin hydrolyis, gelatin liquefaction, and production of botulinum neurotoxin. Pulsed-field gel electrophoresis (PFGE) was done as described (*17*). Cases of adult intestinal toxemia botulism were confirmed by detection of *C*. *botulinum* in stool specimens over prolonged periods in conjunction with clinical observations consistent with botulism. One of these cases has been reported separately as a case study (*15*).

## Case Reports

### Case-Patient 1

A 63-year-old woman was admitted to a hospital on November 22, 2006, after a 2-day history of abdominal pain, blurred vision, diarrhea, dysarthria, dysphagia, horizontal binocular diplopia, imbalance, and weakness in the arms and hands. No recent infections, wounds, or antimicrobial drug use were reported. After admission to the hospital, she experienced respiratory arrest, for which intubation and ventilation were required. The patient’s medical history included Crohn disease, bowel surgery, short bowel syndrome, hypertension, breast cancer, nephrolithiasis, and small fiber polyneuropathy (motor function was normal on nerve conduction studies 6 years earlier). A pathology report from 20 years earlier noted acute and chronic inflammation of the patient’s large and small bowels. No acute wounds or punctures were apparent on examination.

The neurologic examination showed a normal level of consciousness and mental state; reduced pupillary light responses; bilateral, symmetrical ophthalmoplegia; bilateral ptosis; bifacial lower motor neuron weakness; moderate proximal and distal symmetrical limb weakness; absent tendon reflexes; and flexor plantar responses. Muscle tone was decreased; neither atrophy nor fasciculation occurred, and sensation was intact. Results of routine blood and urine tests showed only a mild elevation of the serum amylase level. Botulism was strongly suspected. However, other conditions that affect the central nervous system, including the brainstem and cranial nerves, were considered. Results of tests for syphilis, Lyme disease, West Nile virus infection, acetylcholine receptor antibodies, and IgG against GQ1b were negative.

Results of cerebrospinal fluid studies, including cytology and viral PCR studies, were also normal. A magnetic resonance imaging scan of the brain had unremarkable findings. Electrodiagnostic studies showed mildly reduced compound motor action potential amplitudes and normal sensory responses. Posttetanic facilitation was marked, with a 700% increase in compound motor action potential amplitudes. Electromyography showed that lower limb muscles were electrically silent. Enema fluid, stool, and gastric aspirate specimens were positive for type A botulinum neurotoxin and viable *C*. *botulinum* type A spores, with organisms persisting in stool for 61 days. Botulinum antitoxin was administered in the evening of November 23, 2006.

The environmental investigation found that the patient ate a limited diet of tea, bagels, peanut butter, occasional honey, and weekly Chinese take-out food. An opened jar of peanut butter from the patient’s residence yielded positive test results for *C. botulinum* type A spores, containing ≈14 *C. botulinum* spores/kg.

Both heated and nonheated samples from the enema fluid were positive for *C. botulinum* type A, indicating the presence of spores. Analysis of clinical and peanut butter isolates by PFGE indicated identical PFGE patterns (Figure).

**Figure:** Results of pulsed-field gel electrophoresis of samples from case-patient 1, who had intestinal toxemia botulism, Ontario, Canada, 2006–2008. Lanes 1, 3: *Clostridium botulinum* type A isolated from enema sample; lanes 2, 4: *C. botulinum* type A isolated from peanut butter obtained from residence of patient. Samples in lanes 1 and 2 were digested with *Sma*I; samples in lanes 3 and 4 were digested with *Xho*I.

The patient was still mechanically ventilated as of December 15, 2006, and required regular sedation. Once sedation wore off, the patient experienced random, spastic-like, nonpurposeful movements of all extremities. Her condition improved over the next 6 months, and she could walk by the time she was discharged.

### Case-Patient 2

Bulbar symptoms developed in a 50-year-old woman with ophthalmoparesis on February 7, 2007, and her symptoms worsened until she was admitted to the hospital on February 10, 2007, with complete ophthalmoplegia, dysphagia, and quadriparesis. The patient’s medical history included a diagnosis of Crohn disease in 1979 and 4 previous bowel resections with ileocolonic anastomosis, complicated by enterocutaneous fistulas (in 1986, 1995, 2001, and 2002). She had no wounds, and no chest or abdominal pains were reported before the onset of symptoms. The patient experienced oropharyngeal discomfort and difficulty speaking but was able to respond to questions with a yes or no. A repeat computed tomography scan was carried out, and results were normal. Lumbar puncture was conducted for full studies, including tests for West Nile virus. Over the next 24 hours, she had a decreased level of consciousness, a depressed Glasgow coma scale score, tachycardia, and difficulty breathing, with eventual respiratory failure requiring ventilation support.

The neurologic examination showed minimal orbicularis oculi contractions and quadriparesis with normal reflexes. The differential diagnoses being considered for this case-patient included the following: viral encephalitis, meningitis, brain stem lesion, myasthenia gravis, and infectious agents, specifically, *C. botulinum* and enteric organisms linked to Guillain-Barré syndrome, such as *Campylobacter*, *Salmonella*, and *Shigella* spp. The initial assessment by the consulting neurology team was that of Miller Fischer variant of Guillain-Barré syndrome and Eaton-Lambert syndrome or acute motor axonal neuropathy. Electromyography studies showed myopathic-like motor unit action potential. Results of a Tensilon test were negative. Additional tests, including a test for antibody against acetylcholine, and repeat electromyography studies with high frequency stimulation were negative (no incremental response and a negative acetylcholine antibody test results), which indicated that myasthenia gravis and Eaton-Lambert syndrome were unlikely. A presumptive diagnosis of botulism from intestinal toxemia, based in part on the patient’s history of Crohn disease and previous bowel surgeries, was made on February 13, 2007. The patient received botulinum antitoxin on February 23, 2007. She remained dependent on a ventilator for 5 months and had a percutaneous endoscopic gastrostomy tube for feeding, leading to line sepsis on 2 occasions, ventilator-assisted pneumonia, and a tracheostomy.

The patient’s food history included consumption of toast with peanut butter, coffee, chicken soup, and home-prepared beef tacos with grated cheese, lettuce, tomatoes, and salsa. Before the onset of symptoms, the patient consumed peanut butter daily. An opened jar of peanut butter from the home yielded positive test results for *C. botulinum* type A.

Of 7 serum samples, the first sample was positive for botulinum neurotoxin; however, the quantity was insufficient for serotype determination. Subsequent serum sample test results were negative for botulinum neurotoxin. Type A botulinum toxin was detected in the first stool specimen, which was collected on February 21, 2007. All subsequent stool specimen test results were negative for botulinum toxin. Test results for 5 of 8 stool specimens were positive for either *C. botulinum* type A or type B spores over 56 days. Three subsequent stool samples collected in May 2007 yielded negative test results for *C. botulinum*.

The patient was transferred to a rehabilitation hospital on June 17, 2007, where she required nightly ventilation support and received therapy for swallowing and speech as well as physiotherapy and occupational therapy. In September 2007, ventilator support was gradually withdrawn; the patient learned to eat on her own and was discharged, but she continued to receive outpatient therapy for an additional 3 months. During a follow-up visit in June 2009, the patient was walking independently, and a respiratory evaluation showed normal ventilatory capacity of forced expiratory volume of 2.58 L, 99% of predicted normal inspiratory muscle strength, and only minimal expiratory muscle weakness.

### Case-Patient 3

A 45-year-old man with no known history of gastrointestinal problems and otherwise healthy was admitted to the hospital on January 8, 2008, with a distended abdomen and blurry vision (*15*). The patient’s abdomen continued to distend, and bowel sounds were diminished despite nasogastric suctioning. No recent infections or antimicrobial drug therapy were noted. The distended abdomen signaled a possible bowel obstruction, and scans confirmed the presence of an obstruction with unknown cause. A neurologic consultation was necessary after 5 days to assess the patient’s worsening neurologic symptoms. Guillain-Barré syndrome and myasthenia gravis were initially considered, but results of a nerve conduction study were classic for botulism. Botulism was suspected on January 14, 2008, on the basis of neurophysiologic findings, coupled with the clinical observations suggestive of botulism.

Clinical samples were subsequently sent for analysis, and through consultation with the physician, neurologist, and public health practitioners, it was determined that the patient would still benefit from antitoxin, even though it had been more than 24 hours since the onset of symptoms. Antitoxin was administered the evening of January 14, 2008. The patient remained hospitalized until February 27, 2008, when clinical symptoms improved. *C. botulinum* type B was detected in the patient’s stool at weeks 2 and 8 after the onset of symptoms. Botulinum neurotoxin was not detected in the stool extracts. Resulting of testing of both heated samples were positive for *C. botulinum* type B, indicating the presence of spores in the sample. Two subsequent fecal samples at weeks 11 and 15 yielded negative test results for *C. botulinum*. The persistence of *C. botulinum* type B over a period of 41 days supported the diagnosis of intestinal toxemia botulism. The patient had a history of eating primarily commercial canned food, although no specific types were reported, and none of the patient’s contacts were reported to be ill.

## Conclusions

Diagnosis of intestinal toxemia botulism was determined through repeated detection of botulinum neurotoxins, viable *C. botulinum,* or both in clinical samples over extended periods. Colonization with *C. botulinum* persisted in case-patients 1, 2, and 3 for 61, 56, and 41 days, respectively. In addition to the persistence of organisms in stool samples, the diagnoses were supported by the presence of clinical features compatible with botulism (Table). In previously published reports, *C. botulinum* was present in stool specimens for periods ranging from 2 to 119 days (*9*). Two additional cases of intestinal toxemia botulism in adults were identified in close proximity and time to the 3 cases reported here; however, consent was not obtained to publish the findings pertaining to these cases.

**Table:** Clinical summary of 3 intestinal toxemia botulism cases in adults, Ontario, Canada, 2006–2008

Case-patient no.	Age, y/sex	Dates admitted to hospital and discharged	Clinical findings	Gastrointestinal history	*Clostridium botulinum* serotype	No. days stool sample positive for C. botulinum*	Supportive care; antitoxin; administration date
1	63/F	2006 Nov 22	Abdominal pain, diarrhea, dysphagia, diplopia, weakness	Crohn disease, short bowel syndrome, remote bowel surgery	A	61	Intubation and ventilation; bivalent type A and B, monovalent type E; 2006 Nov 23
2	50/F	2007 Feb 8; 2007 Sep	Dysphagia, double vision, weakness, difficulty breathing	Crohn disease, bowel resections	A and B	56	Intubation and ventilation; bivalent type A and B, monovalent type E; 2007 Feb 23
3	45/M	2008 Jan 8; 2008 Feb 27	Abdominal distension and obstruction, aphonia, diplopia, dry mouth, dysphagia, dysphonia, ptosis	Unremarkable	B	41	Bivalent type A and B, monovalent type E; 2008 Jan 14

*Total number of days that *C. botulinum* spores or neurotoxins were detected in clinical samples.

Intestinal toxemia botulism in adults is described in detail in earlier reports (*3*), and Arnon summarized 10 previous cases (*2*). Since the summary of Arnon, 3 cases have been reported in Italy (*11*), and a case occurred in Japan in 1999 (*13*). Previously cited risk factors for intestinal toxemia botulism in adults include structural and functional gastrointestinal abnormalities, including Crohn disease (*8*), surgical alterations of the intestine (*6**,**9*), Meckel diverticulum (*12**,**18*), and modified intestinal flora caused by prolonged antimicrobial drug therapy (*6**–**10**,**13*). Two of the patients described in this report had Crohn disease and previous bowel surgery. In addition, case-patient 1 also had short bowel syndrome. Unlike the first 2 case-patients, case-patient 3 was previously healthy and had no history of gastrointestinal problems. Three other published reports of intestinal toxemia botulism in adults occurred in patients with no preexisting medical conditions or antimicrobial drug use reported. These cases occurred in Kentucky, USA (*9*); Iceland (*9*); and Japan (*13*). The initial symptoms of acute abdominal distension and small bowel obstruction experienced by case-patient 3 are uncommon and suggest that the differential diagnosis for small bowel obstruction in an otherwise healthy patient should include botulism (*15*).

Several factors could lead to underdiagnosis of intestinal toxemia botulism. Symptoms of botulism are similar to other conditions affecting the central nervous system, and botulism may be misdiagnosed as Guillain-Barré syndrome, myasthenia gravis, Eaton-Lambert syndrome, stroke syndromes, or tick paralysis (*1**,**19*). Similar to botulism in infants, cases of intestinal toxemia botulism in adults occur sporadically and may be missed unless the physician has seen a case of botulism previously. Two cases of foodborne botulism, both attributed to carrot juice, had been identified in the same region only 2 months before the first case of intestinal toxemia botulism described here (*20*), perhaps contributing to a period of higher awareness of botulism cases.

Given that intestinal toxemia botulism in adults is rare, the identification of multiple cases in a small geographic area in a short period is unusual. Two cases of intestinal toxemia botulism in adults, caused by *C. butyricum* which produced type E neurotoxin, were reported in Italy (*11**,**12*). The cases in Italy occurred in 2 communities ≈30 km apart and were separated by 12 months (*12*).

Two of the case-patients described in our report had Crohn disease and prior bowel surgery. Such conditions are consistent with previously published cases, providing further evidence that host susceptibility may play a substantial role in this disease. Although intestinal toxemia botulism is a rare disease worldwide, it is a serious disease that causes substantial illness and high health care costs. Public health practitioners and physicians investigating suspected botulism cases should thoroughly consider the patient’s medical history, particularly for underlying gastrointestinal conditions that may make them susceptible to intestinal toxemia botulism.

## References

[1] Botulism SJ. Clin Infect Dis. 2005;41:1167–73. 10.1086/44450716163636

[2] Arnon SS. Botulism as an intestinal toxemia. In: Blaser MJ, Smith PD, Ravdin JI, Greenberg HB, Guerrant RL, editors. Infections of the gastrointestinal tract. New York: Raven Press; 1995. p. 257–71.

[3] Bradley WG, Shahani BT, Hyslop NE. Case records of the Massachusetts General Hospital. Weekly clinicopathological exercises. Case 48-1980—rapidly progressive neurologic disorder following gastrointestinal symptoms. N Engl J Med. 1980;303:1347–55.6253784

[4] Case 48-1980: botulism. N Engl J Med. 1981;304:789–91. 10.1056/NEJM1981032630413187464893

[5] Bartlett JC. Infant botulism in adults. N Engl J Med. 1986;315:254–5. 10.1056/NEJM1986072431504113523249

[6] Chia JK, Clark JB, Ryan CA, Pollack M. Botulism in an adult associated with food-borne intestinal infection with *Clostridium botulinum.* N Engl J Med. 1986;315:239–41. 10.1056/NEJM1986072431504073523248

[7] Freedman M, Armstrong RM, Killian JM, Boland D. Botulism in a patient with jejunoileal bypass. Ann Neurol. 1986;20:641–3. 10.1002/ana.4102005163789680

[8] Griffin PM, Hatheway CL, Rosenbaum RB, Sokolow R. Endogenous antibody production to botulinum toxin in an adult with intestinal colonization botulism and underlying Crohn’s disease. J Infect Dis. 1997;175:633–7. 10.1093/infdis/175.3.6339041335

[9] McCroskey LM, Hatheway CL. Laboratory findings in four cases of adult botulism suggest colonization of the intestinal tract. J Clin Microbiol. 1988;26:1052–4.329023410.1128/jcm.26.5.1052-1054.1988PMC266519

[10] McCroskey LM, Hatheway CL, Woodruff BA, Greenberg JA, Jurgenson P. Type F botulism due to neurotoxigenic *Clostridium baratii* from an unknown source in an adult. J Clin Microbiol. 1991;29:2618–20.177427210.1128/jcm.29.11.2618-2620.1991PMC270386

[11] Fenicia L, Anniballi F, Aureli P. Intestinal toxemia botulism in Italy, 1984–2005. Eur J Clin Microbiol Infect Dis. 2007;26:385–94. 10.1007/s10096-007-0301-917516104

[12] Fenicia L, Franciosa G, Pourshaban M, Aureli P. Intestinal toxemia botulism in two young people, caused by *Clostridium butyricum* type E. Clin Infect Dis. 1999;29:1381–7. 10.1086/31349710585782

[13] Kobayashi H, Fujisawa K, Saito Y, Kamijo M, Oshima S, Kubo M, A botulism case of a 12-year-old girl caused by intestinal colonization of *Clostridium botulinum* type Ab. Jpn J Infect Dis. 2003;56:73–4.12824692

[14] Sonnabend WF, Sonnabend OA, Grundler P, Ketz E. Intestinal toxicoinfection by *Clostridium botulinum* type F in an adult: case associated with Guillain-Barré syndrome. Lancet. 1987;i:357–61. 10.1016/S0140-6736(87)91729-62880164

[15] Spiegelman J, Cescon DW, Friedman Y, Mazza BV, Austin JW, Rachlis A, Bowel loops and eyelid droops. CMAJ. 2008;179:927–9. 10.1503/cmaj.08065118936457PMC2565713

[16] Austin JW, Sanders G. Detection of *Clostridium botulinum* and its toxins in suspect foods and clinical specimens. Compendium of analytical methods 2009 [cited 2011 Nov 15]. http://www.hc-sc.gc.ca/fn-an/alt_formats/hpfb-dgpsa/pdf/res-rech/mfhpb16-eng.pdf

[17] Leclair D, Pagotto F, Farber JM, Cadieux B, Austin JW. Comparison of DNA fingerprinting methods for use in investigation of type E botulism outbreaks in the Canadian Arctic. J Clin Microbiol. 2006;44:1635–44. 10.1128/JCM.44.5.1635-1644.200616672387PMC1479196

[18] Schechter R, Arnon SS. Commentary: where Marco Polo meets Meckel: type E botulism from *Clostridium butyricum.* Clin Infect Dis. 1999;29:1388–93. 10.1086/31356410585783

[19] St. Louis ME, Peck SHS, Bowering D, Morgan GB, Blatherwick J, Banerjee S, Botulism from chopped garlic: delayed recognition of a major outbreak. Ann Intern Med. 1988;108:363–8.334167310.7326/0003-4819-108-3-363

[20] Sheth AN, Wiersma P, Atrubin D, Dubey V, Zink D, Skinner G, International outbreak of severe botulism with prolonged toxemia caused by commercial carrot juice. Clin Infect Dis. 2008;47:1245–51. 10.1086/59257418834318

# Review article: Intestinal Toxemia Botulism in 3 Adults, Ontario, Canada, 2006–2008

**DOI:** 10.3201/eid1801.110533

**Authors:** Yolanda D. Sheppard, Dean Middleton, Yvonne Whitfield, Felix Tyndel, Shariq Haider, Jamie Spiegelman, Richard H. Swartz, Mark P. Nelder, Stacey L. Baker, Lisa Landry, Ross MacEachern, Sherri Deamond, Lorrie Ross, Garth Peters, Michelle Baird, David Rose, Greg Sanders, John W. Austin

**Affiliations:** Ministry of Health and Long-Term Care, Toronto, Ontario, Canada (Y.D. Sheppard, Y. Whitfield, M.P. Nelder, S.L. Baker);; Ontario Agency for Health Protection and Promotion, Toronto (D. Middleton);; Scarborough Hospital–Birchmount, Toronto (F. Tyndel);; McMaster University Medical Center, Hamilton, Ontario, Canada (S. Haider);; Humber River Regional Hospital, Toronto (J. Spiegelman);; Sunnybrook Health Sciences Centre, Toronto (R.H. Swartz);; Public Health Agency of Canada, Guelph, Ontario, Canada (L. Landry);; Durham Region Health Department, Whitby, Ontario, Canada (R. MacEachern, S. Deamond);; Niagara Region Public Health, Thorold, Ontario, Canada (L. Ross);; Toronto Public Health, Toronto (G. Peters);; Hamilton Public Health Services, Hamilton (M. Baird);; The Scarborough Hospital, Toronto (D. Rose);; Health Canada, Ottawa, Ontario, Canada (G. Sanders, J.W. Austin)

## Earning CME Credit

To obtain credit, you should first read the journal article. After reading the article, you should be able to answer the following, related, multiple-choice questions. To complete the questions (with a minimum 70% passing score) and earn continuing medical education (CME) credit, please go to www.medscape.org/journal/eid. Credit cannot be obtained for tests completed on paper, although you may use the worksheet below to keep a record of your answers. You must be a registered user on Medscape.org. If you are not registered on Medscape.org, please click on the New Users: Free Registration link on the left hand side of the website to register. Only one answer is correct for each question. Once you successfully answer all post-test questions you will be able to view and/or print your certificate. For questions regarding the content of this activity, contact the accredited provider, CME@medscape.net. For technical assistance, contact CME@webmd.net. American Medical Association’s Physician’s Recognition Award (AMA PRA) credits are accepted in the US as evidence of participation in CME activities. For further information on this award, please refer to http://www.ama-assn.org/ama/pub/category/2922.html. The AMA has determined that physicians not licensed in the US who participate in this CME activity are eligible for *AMA PRA Category 1 Credits*™. Through agreements that the AMA has made with agencies in some countries, AMA PRA credit may be acceptable as evidence of participation in CME activities. If you are not licensed in the US, please complete the questions online, print the certificate and present it to your national medical association for review.

## Article Title: Intestinal Toxemia Botulism in 3 Adults, Ontario, Canada, 2006–2008

## CME Questions

**1. You are seeing a 52-year-old woman with abdominal pain as well as neurologic complaints, and you consider whether she might have adult intestinal toxemia botulism. Which of the following statements regarding adult intestinal toxemia botulism is most accurate?**A. It produces an ascending rigid paralysisB. It occurs after direct ingestion of neurotoxin but not sporesC. There have been fewer than 20 cases reported over the last 30 yearsD. The primary infecting organism is *Clostridium butyricum***2. In working through the differential diagnosis for this patient, what should you consider as an important risk factor for adult intestinal toxemia botulism?**A. Regular use of proton pump inhibitorsB. Recent infection with *Salmonella* speciesC. The presence of Crohn diseaseD. Chronic laxative use**3. Which of the following statements regarding the clinical presentation of patients with adult intestinal toxemia botulism in the current case series is most accurate?**A. All patients presented with ocular symptoms and signsB. All patients presented with fasciculationsC. The most common source of botulism was undercooked meatD. The most common source of botulism was fresh vegetables**4. Which of the following statements regarding the workup and prognosis of cases of adult intestinal toxemia botulism in the current case series is most accurate?**A. All patients had positive stool testing for more than 30 daysB. Nerve conduction studies were not helpful in the workup of any caseC. No patient required mechanical ventilationD. All patients died as a result of complications of adult intestinal toxemia botulism

## Activity Evaluation

**Table:** 

1. The activity supported the learning objectives.				
Strongly Disagree				Strongly Agree
1	2	3	4	5
2. The material was organized clearly for learning to occur.				
Strongly Disagree				Strongly Agree
1	2	3	4	5
3. The content learned from this activity will impact my practice.				
Strongly Disagree				Strongly Agree
1	2	3	4	5
4. The activity was presented objectively and free of commercial bias.				
Strongly Disagree				Strongly Agree
1	2	3	4	5
